# A case of psoriasiform eruption developed during imatinib therapy^☆^

**DOI:** 10.1016/j.abd.2023.12.006

**Published:** 2024-08-06

**Authors:** Yukina Watanabe, Tomoko Hiraiwa, Mikio Ohtsuka, Toshiyuki Yamamoto

**Affiliations:** Department of Dermatology, Fukushima Medical University, Fukushima, Japan

Dear Editor,

Imatinib is the standard first-line systemic treatment for chronic myeloid leukemia and Gastrointestinal Stromal Tumor (GIST), targeting BCR-ABL and c-KIT tyrosine kinases, respectively. Imatinib-induced eruptions can present with a variety of skin manifestations, but cases with psoriasis/psoriasiform eruptions are rare. We herein report one such case with a review of the literature.

A 69-year-old man was referred to our department with a psoriasiform eruption. He had been treated with imatinib for GIST for the previous 2 years. Two months after the start of imatinib treatment at 400 mg/day, a rash appeared. Physical examination revealed red papules with scales of 2–4 mm in size on the trunk and limbs. In addition, the buttocks had a number of scaly erythema resembling psoriasis (Fig. 1A). A skin biopsy was performed from the scaly erythema of the lower leg (Fig. 1B). Histological findings revealed mild epidermal proliferation with parakeratosis, and subepidermal dilatation of capillaries with perivascular infiltration of mononuclear cells, containing eosinophils (Fig. 1C). Blood test revealed that the patient's eosinophils were elevated between 10% and 15%. Based on the course of the disease, a diagnosis of drug eruption caused by imatinib was made. Treatment was begun with oral antihistamine and topical corticosteroids, and the skin rash improved (Fig. 1D). During the treatment period, imatinib was continued without dose reduction.

**Fig. 1:**
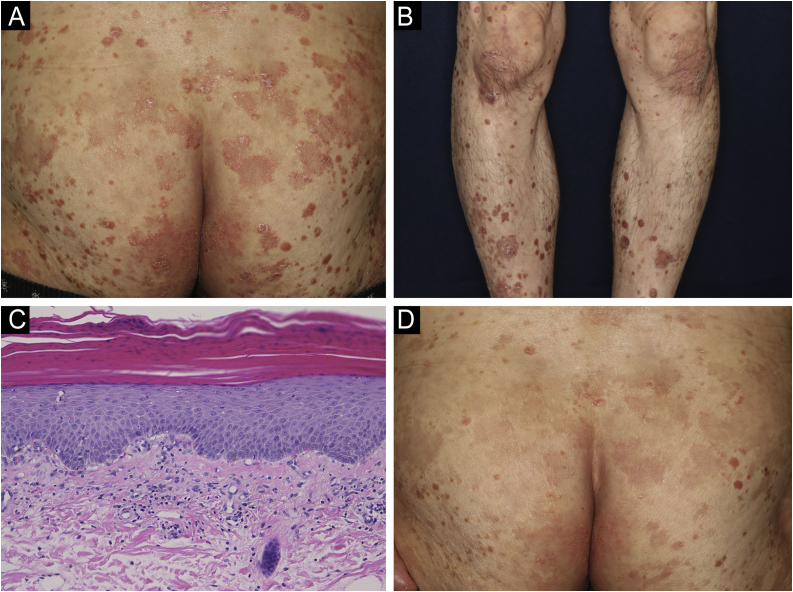
(A) Psoriasiform lesions on the buttocks. (B) Similar lesions on the lower legs. (C) Histological findings revealed slight parakeratosis with a slightly flattened epidermis. Inflammatory cell infiltration was observed around blood vessels and stroma in the superficial dermis, accompanied by red blood cell extravasation. The inflammatory cells were eosinophils as well as lymphocytes and histiocytes. (Hematoxylin & eosin, ×200). (D) After treatment, the skin rash was partially improved.

Cutaneous reactions are the most commonly reported nonhematological side effects, occurring in 9.5%–69% of patients.^1^ Maculopapular or erythematous eruptions, edema, and periorbital edema are the most common adverse events observed. Severe drug eruptions such as Stevens-Johnson syndrome and toxic epidermal necrolysis have also been reported. Imatinib potentially affects immune cells directly, and several cases showed improvement in psoriasis after the introduction of imatinib.^2^, ^3^ By contrast, exacerbation of psoriasis or do novo development of psoriasiform rash have rarely been reported. As a pathogenetic mechanism, it has been reported that imatinib treatment reduced CD4+CD25+FoxP3+ regulatory T-cell (Treg) frequency and decreased immunosuppressive function.^4^ Therefore, reduced activation of Tregs by imatinib may contribute to the development of psoriasis or psoriasiform eruptions. It has also been reported that psoriasis/psoriasiform eruptions are generally less severe, due to effector T-cell inhibition by imatinib.^2^

We reviewed the English literature on PubMed from 2002 to 2022, using the keywords “imatinib” and “psoriasis”. The results of our search revealed eight cases,^2^, ^3^, ^5^, ^6^, ^7^, ^8^, ^9^, ^10^ the details of which are summarized in Table 1. Eight cases were reported to date in which imatinib caused psoriasis exacerbation, new onset, or even improvement. The average age at occurrence was 51.1 years old, with a male predominance (6:2 men to women ratio). The duration of onset ranged from a few weeks to several months. There were seven patients with pre-existing psoriasis, and the remaining patients had newly onset psoriasis symptoms. Since the present case was also a new onset psoriasiform eruption, we focused on the previous case of the same type. There were differences in histological findings and the course of the disease. The histopathological features in the previous case were neutrophilic scale crust and loss of the granular cell layer.^5^ By contrast, the histopathological features in the present case were atypical for psoriasis, in that slight parakeratosis, no clear epidermal proliferation, and the absence of neutrophilic microabscess below the corneal layers. The patient in the previous case was switched to a different drug,^5^ but the patient in the present case was able to continue with imatinib.

**Table 1:** Case of imatinib and psoriasis associated.

	Age, Sex	Imatinib dose	Rash	History of psoriasis	Duration	Therapy	Treatment outcome
1^6^	52, M	400 mg/day	Exacerbation	+	2 months	Topical corticosteroid, calcipotriol ointment, Resumption of imatinib at 200 mg/day	Improved
2^7^	55, M	400 mg/day	Exacerbation	+	2 months	Topical corticosteroid and vitamin D analogues, continued on imatinib.	Persisted
3^8^	62, F	400 mg/day	Exacerbation	+	4 weeks	Discontinuation of imatinib, resumption of imatinib at 400 mg/day, MTX12.5 mg/week	Improved
4^9^	63, M	400 mg/day	Exacerbation	+	3 weeks	Discontinuation, narrow-band UVB	Improved
5^10^	57, M	400 mg/day	Exacerbation	+	Unknown	Discontinuation, resumption of imatinib at 200 mg/day, vitamin D3 ointment	Improved
6^5^	21, M	400 mg/day	New onset	−	5 months	Discontinuation, narrow-band UVB	Improved
7^2^	35, M	400 mg/day	Improvement	+	1 month	Undescribed	Undescribed
8^3^	64, M	400 mg/day	Improvement	+	2 weeks	Undescribed	Undescribed
Present case	69, M	400 mg/day	New onset	–	2 months	Reduction of imatinib to 300 mg/day, topical corticosteroid	Improved

In the present report, we described a rare case of de novo development of psoriasiform eruption under imatinib treatment. Imatinib-induced psoriasis/psoriasiform eruption takes several months to develop and appears dose-dependent. Consequently, it is thought to be related to pharmacological effects rather than an allergic mechanism. In the present case, it had taken 2 months for the rash to develop. However, the rash improved without dose-reduction of imatinib. Furthermore, the patient’s eosinophils were elevated after the start of imatinib but had improved after the end of imatinib. Also, eosinophil infiltration was marked in the histological findings. Thus, we suggested that an allergic mechanism or other pharmacological effects may be involved.

## Financial support

None declared.

## Authors’ contributions

Yukina Watanabe: Approval of the final version of the manuscript; preparation and writing of the manuscript.

Tomoko Hiraiwa: Approval of the final version of the manuscript; intellectual participation in propaedeutic and/or therapeutic management of studied cases.

Mikio Ohtsuka: Approval of the final version of the manuscript; intellectual participation in propaedeutic and/or therapeutic management of studied cases.

Toshiyuki Ymamoto: Manuscript critical review; approval of the final version of the manuscript.

## Conflicts of interest

None declared.
